# Supplementary material for: Three metabolic pathways replenishing the one-carbon pool collectively support growth and virulence of Listeria monocytogenes
Source: eLife. 2026 Aug 19;14:RP109227. doi: 10.7554/eLife.109227 (PMC13489624; doi:10.7554/eLife.109227)
Supplement: Peer review history

# Editor report: eLife Assessment

**DOI:** 10.7554/eLife.109227.3.sa0

**Authors:** Vinay Kumar Nandicoori

**Affiliations:** National Institute of Immunology India

**Keywords:** Solid, Valuable

One-carbon tetrahydrofolate metabolism plays a crucial role in producing essential metabolic intermediates. In this **valuable** study, the authors employ a **solid** genetics-based approach to demonstrate that three distinct metabolic pathways are essential for synthesizing 1C-tetrahydrofolates (1C-THF). Disrupting any of these pathways impairs both growth and virulence.

# Referee report: Reviewer #1 (Public review)

**DOI:** 10.7554/eLife.109227.3.sa1

Summary:

This study identifies three redundant pathways-glycine cleavage system (GCS), serine hydroxymethyltransferase (GlyA), and formate-tetrahydrofolate ligase/FolD-that feed the one-carbon tetrahydrofolate (1C-THF) pool essential for Listeria monocytogenes growth and virulence. Reactivation of the normally inactive fhs gene rescues 1C-THF deficiency, revealing metabolic plasticity and vulnerability for potential antimicrobial targeting.

Strengths:

(1) Novel evolutionary insight-Reversible reactivation of a pseudogene (fhs) shows adaptive metabolic plasticity, relevant for pathogen evolution.

(2) They systematically combine targeted gene deletions with suppressor screening to dissect the folate/one-carbon network (GCS, GlyA, Fhs/FolD).

# Referee report: Reviewer #3 (Public review)

**DOI:** 10.7554/eLife.109227.3.sa2

Summary:

In this study, Freier et al., demonstrate that 3 distinct metabolic pathways are critical for the synthesis of 1C-THF, a metabolite that is crucial for the growth and virulence of Listeria monocytogenes. Using an elegant suppressor screen, they also demonstrate the hierarchical importance of these metabolic pathways with respect to the biosynthesis of 1C-THF.

Strengths:

This study uses elegant bacterial genetics to confirm that 3 distinct metabolic pathways are critical for 1C-THF synthesis in L. monocytogenes and lack of either one of these pathways compromises bacterial growth and virulence. The study uses a combination of in vitro growth assays, macrophage-CFU assays and murine infection models to demonstrate this.

Comments on revisions:

The revised manuscript is improved, and the additional genetic experiments provide further support for the proposed metabolic model. However, the central conclusion is not fully established without direct measurement of 1C-THF levels. While I appreciate the authors' explanation regarding the technical limitations, quantitative metabolite measurements (e.g., by mass spectrometry) would have provided much stronger evidence linking the genetic perturbations to altered 1C-THF pools.

# Author comment: Author response

**DOI:** 10.7554/eLife.109227.3.sa3

**Authors:** Sandra Freier, Sarah Frentzel, Moritz Müller, Susan Scheffler, Sabrina Wamp, Tim Engelgeh, Janina Döhling, Dunja Bruder, Sascha Kahlfuss, Sven Halbedel

**Affiliations:** Otto-von-Guericke University Magdeburg Magdeburg Germany; Otto-von-Guericke University Magdeburg Magdeburg Germany; Robert Koch Institute Wernigerode Germany; Robert Koch Institute Wernigerode Germany; Robert Koch Institute Wernigerode Germany; Robert Koch Institute Wernigerode Germany; Robert Koch Institute Wernigerode Germany; Otto-von-Guericke University Magdeburg Magdeburg Germany; Otto-von-Guericke University Magdeburg Magdeburg Germany; Robert Koch Institute Wernigerode Germany

The following is the authors’ response to the original reviews.

**Public Reviews:**

**Reviewer #1 (Public review):**

Summary:

This study identifies three redundant pathways-glycine cleavage system (GCS), serine hydroxymethyltransferase (GlyA), and formate-tetrahydrofolate ligase/FolD-that feed the one-carbon tetrahydrofolate (1C-THF) pool essential for Listeria monocytogenes growth and virulence. Reactivation of the normally inactive fhs gene rescues 1C-THF deficiency, revealing metabolic plasticity and vulnerability for potential antimicrobial targeting

Strengths:

(1) Novel evolutionary insight - reversible reactivation of a pseudogene (fhs) shows adaptive metabolic plasticity, relevant for pathogen evolution.

(2) They systematically combine targeted gene deletions with suppressor screening to dissect the folate/one-carbon network (GCS, GlyA, Fhs/FolD).

Weaknesses:

(1) The study infers 1C-THF depletion mostly genetically and indirectly (growth rescue with adenine) without direct quantification of folate intermediates or fluxes. Biochemical confirmation, LC-MS-based metabolomics of folates/1C donors, or isotopic tracing would strengthen mechanistic claims.

We agree with the reviewer that quantification of 1C-THF intermediates would strengthen our conclusions. However, the chemical methodologies to extract folates from *L. monocytogenes* are not established in our lab. Moreover, quantification of C1-substituted folates requires comprehensive biochemical and analytical expertise that we also do not have and which we cannot cover though co-operations. However, to further strengthen our arguments, we have introduced an experiment in the updated manuscript that demonstrates synthetic lethality of a Δ*gcvPAB* Δ*glyA* mutant with a deletion of *fold* (Fig. 7A). This gene encodes 5,10-methylene-tetrahydrofolate dehydrogenase/ 5,10-methylene-tetrahydrofolate cyclohydrolase, which is the third enzyme involved in N5, N10-methylene-THF generation next to GlyA and GcvPBA. Synthetic lethality of a Δ*gcvPAB* Δ*glyA* double mutant with a *fold* deletion is best explained by GcvPAB and GlyA also feeding the N5,N10-methylene-THF pool.

(2) In multiple result sections, the authors report data from technical triplicates but do not mention independent biological replicates (e.g., Figure 2C, Figure 4A-B, Figure 6D). In addition, some results mention statistical significance but without a detailed description of the specific statistical tests used or replicates, such as Figure 2A-C, Figure 2E, and Figure 2G-I.

We thank the reviewer for this helpful comment. Experiments were usually repeated three independent times, with each repetition including three technical replicates. Mean values and standard deviations were usually calculated from the technical replicates of a representative run. Statistical significance was calculated using *t*-tests for pairwise comparisons or *t*-tests using Bonferroni-Holm correction for multiple comparisons. We made sure that this is explicitly explained for each experiment in the figure legends. Wherever other calculations were used, we also clarified this in the legends.

**Reviewer #2 (Public review):**

Summary:

The manuscript by Freier et al examines the impact of deletion of the glycine cleavage system (GCS) GcvPAB enzyme complex in the facultative intracellular bacterial pathogen Listeria monocytogenes. GcvPAB mediates the oxidative decarboxylation of glycine as a first step in a pathway that leads to the generation of N5, N10-methylene-Tetrahydrofolate (THF) to replenish the 1-carbon THF (1C-THF) pool. 1C-THF species are important for the biosynthesis of purines and pyrimidines as well as for the formation of serine, methionine, and N-formylmethionine, and the authors have previously demonstrated that gcvPAB is important for bacterial replication within macrophages. A significant defect for growth is observed for the gcvPAB deletion mutant in defined media, and this growth defect appears to stem from the sensitivity of the mutant strain to excess glycine, which is hypothesized to further deplete the 1C-THF pool. Selection of suppressor mutations that restored growth of gcvPAB deletion mutants in synthetic media with high glycine yielded mutants that reversed stop codon inactivation of the formatetetrahydrofolate ligase (fhs) gene, supporting the premise that generation of N10-formyl-THF can restore growth. Mutations within the folk, codY, and glyA genes, encoding serine hydroxymethyltransferase, were also identified, although the functional impact of these mutations is somewhat less clear. Overall, the authors report that their work identifies three pathways that feed the 1C-THF pool to support the growth and virulence of L. monocytogenes and that this work represents the first example of the spontaneous reactivation of a L. monocytogenes gene that is inactivated by a premature stop codon.

Strengths:

This is an interesting study that takes advantage of a naturally existing fhs mutant Listeria strain to reveal the contributions of different pathways leading to 1C-THF synthesis. The defects observed for the gcvPAB mutant in terms of intracellular growth and virulence are somewhat subtle, indicating that bacteria must be able to access host sources (such as adenine?) to compensate for the loss of purine and fMet synthesis. Overall, the authors do a nice job of assessing the importance of the pathways identified for 1C-THF synthesis.

Weaknesses:

(1) Line 114 and Figure 1: The authors indicate that the gcvPAB deletion forms significantly fewer plaques in addition to forming smaller plaques (although this is a bit hard to see in the plaque images). A reduction in the overall number of plaques sounds like a bacterial invasion defect - has this been carefully assessed? The smaller plaque size makes sense with reduced bacterial replication, but I'm not sure I understand the reduction in plaque number.

The observation that the Δ*gcvPAB* mutant forms fewer plaques was not our claim, and we have already addressed the possibility of an invasion defect by quantifying bacterial numbers during infection of 3T3 cells. As shown in Fig. 2A, the Δ*gcvPAB* mutant invades 3T3 cells (the same cells used in the plaque formation assays) as efficiently as the wild type but exhibits reduced intracellular growth. Therefore, the plaquing defect is not due to impaired invasion. Furthermore, we also have analyzed the intracellular dissemination of the Δ*gcvPAB* mutant in 3T3 fibroblasts compared to a Δ*actA* mutant by microscopy. This shows that the Δ*gcvPAB* is evenly distributed throughout the infected host cells as the wild type and unlike the Δ*actA* mutant, which forms clusters (Fig. S1). Both experiments indicate that the reduced plaque area results from impaired intracellular growth rather than defects in invasion or cell-to-cell spread. The apparent reduction in plaque numbers in Δ*gcvPAB*-infected 3T3 cells is likely due to a strong reduction in plaque size, with only the largest plaques remaining visible. We have rephrased the relevant section to clarify this point and avoid any potential confusion:

“In agreement with our previous results, only small plaques were formed in 3T3 cells upon infection with the Δ*gcvPAB* mutant (plaque area: 15±19% of wild-type level) and small plaques were also formed by the complemented strain in the absence of IPTG (50±19%).”

(2) Do other Listeria strains contain the stop codon in fhs? How common is this mutation? That would be interesting to know.

We determined the frequency of inactivated *fhs* genes among 30,000 publicly available *L. monocytogenes* genomes. The analysis identified only 10 isolates carrying truncated *fhs* alleles. These isolates fell into two groups: (i) EGD-e and its descendants, and (ii) a cluster of five ST2 food isolates. These findings have been added as a separate results section.

(3) Based on the observation that fhs+ ΔgcvPAB ΔglyA mutant is only possible to isolate in complex media, and fhs is responsible for converting formate to 1C-THF with the addition of FolD, have the authors thought of supplementing synthetic media with formate and assessing mutant growth?

No, we did not test formate supplementation. However, we included additional experiments testing adenine and thymine supplementation (Fig. 6E and 7B). These results show that purine and thymine become limiting in mutants lacking 1C-THF synthesizing pathways.

**Reviewer #3 (Public review):**

Summary:

In this study, Freier et al. demonstrate that 3 distinct metabolic pathways are critical for the synthesis of 1C-THF, a metabolite that is crucial for the growth and virulence of Listeria monocytogenes. Using an elegant suppressor screen, they also demonstrate the hierarchical importance of these metabolic pathways with respect to the biosynthesis of 1C-THF.

Strengths:

This study uses elegant bacterial genetics to confirm that 3 distinct metabolic pathways are critical for 1CTHF synthesis in L. monocytogenes, and the lack of either one of these pathways compromises bacterial growth and virulence. The study uses a combination of in vitro growth assays, macrophage-CFU assays, and murine infection models to demonstrate this.

Weaknesses:

(1) The primary finding of the study is that the perturbation of any of the 3 metabolic pathways important for the synthesis of 1C-THF results in reduced growth and virulence of L. monocytogenes. However, there is no evidence demonstrating the levels of 1C-THF in the various knockouts and suppressor mutants used in this study. It is important to measure the levels of this metabolite (ideally using mass spectrometry) in the various knockouts and suppressor mutants, to provide strong causality.

As already outlined above, we do not have any experimental possibilities to measure 1C-substituted THF in *L. monocytogenes* extracts directly. However, to provide additional evidence for our interpretation that “Three pathways feed the 1C-THF pool…”, we included additional genetic experiments.

The first experiment demonstrates that the growth defect of the *fhs*- Δ*glyA igcvPAB* strain in synthetic medium lacking IPTG—which reflects the synthetic lethality of *fhs* with *glyA* and *gcvPAB*—can be rescued by the addition of adenine (Fig. 6E). This indicates that the *fhs/fold* pathway, GlyA, and the glycine cleavage system are essential due to their combined contribution to purine biosynthesis. This result confirms the metabolic model presented in Fig. 1A and thus supports the hypothesis that all three pathways contribute to 1C-THF biosynthesis.

The second experiment additionally demonstrates synthetic lethality of *gcvPAB* and *glyA* with the *fold* gene. FolD acts downstream of Fhs and is one of the three enzymes synthesizing N5, N10methylene-THF shown in Fig. 1A. The *fold* gene is essential in EGD-e (PMID: 36114002), most likely explained by *fhs* inactivation. However, we were able to delete *fold* in an EGD-e background carrying a reconstituted *fhs* gene and the resulting *fhs*^+^ Δ*fold* strain was as viable as a *fhs*^+^ Δ*glyA* Δ*gcvPAB* strain (Fig. 7A). However, a *fhs*^+^ Δ*fold* Δ*glyA* i*gcvPAB* strain required IPTG for growth in BHI medium (Fig. 7A), indicating that the simultaneous deletion of *glyA* and *gcvPAB* is not tolerated in the absence of *fold*, similar to what is observed in the absence of *fhs*. Notably, this growth defect was not rescued by adenine supplementation (Fig. 7B), but was alleviated by thymine addition, which is also consistent with the metabolic model shown in Fig. 1A.

Even though we are unable to directly demonstrate reduced 1C-THF levels, we hope that these two genetic approaches together with the revised title and heading of the relevant paragraph will, in the reviewers’ eyes, support our hypotheses.

(2) The story becomes a little hard to follow since macrophage-CFU assays and murine infection model data precede the in vitro growth assays. The manuscript would benefit from a reorganization of Figures 2,3, and 4 for better readability and flow of data.

We respectfully disagree with the reviewer. The attenuation of the Δ*gcvPAB* mutant in macrophages and fibroblasts was the primary motivation for further investigating its phenotype. Therefore, we chose to begin the manuscript with results from various virulence studies before presenting the sections that provide mechanistic explanations. In our view, this sequence represents a more logical and coherent way to present the data.

**Recommendations for the authors:**

**Reviewer #1 (Recommendations for the authors):**

(1) Synthetic medium assumptions: LSM "mimics" intracellular limitation but isn't chemically validated against host cytosolic composition. Nutrient availability conclusions could be biased.

This is correct. We added this information:

“LSM broth is a chemically defined medium that contains all components required for growth at defined concentrations, but it has not been chemically validated to reflect host cytosolic conditions…”

(2) Glycine toxicity: The paper introduces the concept of glycine toxicity in ΔgcvPAB mutants. However, the conditions under which glycine becomes toxic could be further elucidated. Why glycine causes toxicity despite being an essential metabolite in other contexts requires a more in-depth mechanistic explanation.

The concept of glycine toxicity in GCS mutants has been described previously by other researchers. We have added further details to better explain glycine toxicity and how it accounts for the growth phenotype of the Δ*gcvPAB* mutant:

“If glycine cannot be catabolized (and 1C-THF cannot be generated) by the GCS due to deletion of *gcvPAB*, glycine might be re-routed to the serine hydroxymethyl transferase GlyA for serine formation, even though this would consume 1C-THF and therefore even further deplete the cell for 1C-THF.” and

“In the complete absence of glycine, growth of the Δ*gcvPAB* mutant was largely unaffected, presumably because glycine cannot be converted to serine by GlyA anymore, thereby conserving the 1C-THF pool.”

(3) Figures:

(a) Figure 1B, scale bar?

A scale bar was added.

(b) Figure 1C, the standard error bars for igcvPAB (both with and without IPTG) are relatively wide, indicating high variability in the data. This suggests that the results in the igcvPAB group are not as consistent as the wild-type (wt) or ΔgcvPAB groups. Please show the original data and perform a statistical test (e.g., t-test or ANOVA).

The original data have been added to Fig. 1B. *t*-test results (with Bonferroni-Holm correction) are now included for all samples.

(c) Figure 2D, scale bar?

These are sections of agar plates. From our point of view, a scale bar does not add relevant information.

(d) Figure 3, please check the labels of the Figure 3 legend. (D) and (E) or A-B?

Thanks, corrected.

(e) Figure 5E, quantification of plaque areas?

The plaque areas were quantified. A blot showing these quantitative data is now presented in Fig. 5F.

**Reviewer #2 (Recommendations for the authors):**

Line 58: There are published studies that indicate that syncytiotrophoblasts are actually resistant to Listeria infection and that it is extravillous trophoblasts that are likely to serve as entry points for Listeria into the placenta (see, for example, Lowe et al, Infect Immun. 2018 Volume 86 Issue 6 e00801-17).

We thank the reviewer for this comment and have removed our statement claiming that syncytiothrophoblasts are the entry point as this is not relevant to the understanding of the work presented here.

**Reviewer #3 (Recommendations for the authors):**

(1) Please mention the number of times experiments were performed as independent biological replicates, wherever applicable.

We added this information to the figure legends wherever this was necessary.

(2) Please provide the details of the type of statistical analysis used for the various graphs, either in the figure legends or in the materials & methods section.

This information was also added to the figure legends wherever it still was missing.

(3) Can the authors comment on how the weight-loss phenotype of animals and the variation in the size of the spleen between animals infected with wild-type and mutants in Figure 2 can be explained without any significant changes in the CFU? Additionally, I did not see details regarding the number of animals used in the murine infection model experiments. Please mention this along with the type of statistical analyses used.

We do not see a contradiction here, as the apparent differences are explained by the distinct time points at which CFU numbers (day 3 post-infection) and spleen size (day 9 postinfection) were measured. Starting from day 8, the difference in weight between animals infected with the wild-type strain and those infected with the Δ*gcvPAB* mutant becomes clear for the first time. At day 3, no significant differences are detected in either CFU numbers or weight. By day 9, when the weight difference has become apparent, differences in spleen size are also observed. To improve clarity, the time points at which each analysis was performed have been added to Fig. 2G and Fig. 2I. The number of infected animals and the type of statistical analysis used are now specified in the figure legend.
